# The Declining Birth-Rate

**Published:** 1908-02-01

**Authors:** 


### The Declining Birth-Rate.

The latest report of the Registrar General on the births, deaths, and marriages in England and Wales in 1906, issued on January 27 of this year, provides a subject for serious reflection to thoughtful people. The birth-rate, which has been steadily falling for the past thirty years, reached its lowest recorded level during 1906. In that year 935,081 births were registered, making a proportion of 27.1 per thousand of the total population, which was estimated at 34,547,016 for England and Wales. This birth-rate is .1 per thousand below that for 1905, and 1.6 per thousand below the average for the previous decade. The highest recorded rate was 36.3 per thousand in 1876, the year following the establishment of civil registration, and since 1876 there has been a steady decline in the birth-rate. Thus, during the fifteen years from 1876 to 1891 it fell from 36.3 to 31.4, a difference of 4.9 per thousand; and during the fifteen years from 1891 it has fallen from 31.4 to 27.1, a difference of 4.3; producing a total decline of 9.2 per thousand for the past thirty years. Moreover, from the Registrar General's provisional estimate for the first three quarters of the year 1907, it would appear that this diminution is still progressing.

Those who regard a falling birth-rate, irrespective of other conditions, satistical or otherwise, as an unmixed national evil, will find only one crumb of comfort in the above figures. Thus, although the proportion of births to the total population has steadily diminished year by year for thirty years, the decrease was greater during the first half of that period than it has been during the second half. Meanwhile the death-rate for England and Wales, which has long been considerably lower than the birth-rate, has fallen during the last fifteen recorded years from 20.2 per thousand in 1891 to 15.4 in 1906; and it will be noted that this decrease of 4.8 per thousand is .5 greater than the decrease in the birth-rate during the same period of fifteen years. It cannot justly be contended that a falling death-rate in any way excuses a corresponding decline in birth-rate; but the first is at least some compensation for the second, and, from the point of view of national and social progress, the two sets of statistics should be considered together.

Another table of proportions, which has a closer connection with the birth-rate, is the marriage-rate, and here we cannot find cause for much satisfaction. Considered alone, the marriage statistics for the past fifteen years or so are not particularly unsatisfactory; they have varied but little, and there has been no marked decline. But this very fact that the marriage-rate is not progressively diminishing is, in our opinion, a matter for serious national concern, when it is laid beside the steadily falling birth-rate. Although about as many people are married every year, they are having fewer children. It would seem that matrimony retains its charms, whilst its obligations are becoming less and less respected. If the latter conclusion is denied, then, in order to explain matters, we must presume that the fertility of our race is diminishing: and of the two conclusions, the latter is no doubt the more disquieting. But there is reason to suppose that national neglect of the reproductive obligations of marriage leads to national impairment of the power of reproduction. Thus, to those who have the future welfare of their country at heart, and who believe, as we do, that fertility is one of the safeguards of national progress and prosperity, the present falling birth-rate (and, considered beside it, the present stationary marriage-rate) is a subject for much uneasiness of mind. It behoves the members of the medical profession, above all others, to study these facts and figures, to face the conclusions which may be drawn from them, and to use their influence in the right direction.

